
JOURNAL INFORMATION
==============================
NLM Title Abbreviation: ScientificWorldJournal
Journal ID: ScientificWorldJournal
NLM Journal ID: 101131163
Journal ID: TSWJ

The Scientific World Journal

ISSN: 2356-6140
EISSN: 1537-744X
Publisher: Wiley

ARTICLE INFORMATION
==============================
PMCID: PMC5900956
PMID: 17704852
DOI: 10.1100/tsw.2007.212
Article ID: 597192
Article version: 1
Subjects: Directions in Science

Syncytin and Cancer Cell Fusions

Larsson Lars-Inge *
Bjerregaard Bolette
Wulf-Andersen Linda
Talts Jan Fredrik
Anatomy and Cell Biology, IBHV, Faculty of Life Sciences, University of Copenhagen, Gronnegaardsvej 7, DK-1809 Frederiksberg C, Denmark
*Lars-Inge Larsson: lail@life.ku.dkAcademic Editor: Martin Goette

Publication date: 2007
Electronic publication date: 2007 Aug 17
Volume: 7
First page: 1193
Last page: 1197
Received 2007 Jul 13; Accepted 2007 Jul 19
Copyright: Copyright © 2007 Lars-Inge Larsson et al.
Copyright year: 2007
License: This is an open access article distributed under the Creative Commons Attribution License, which permits unrestricted use, distribution, and reproduction in any medium, provided the original work is properly cited.
License URL: https://creativecommons.org/licenses/by/3.0/

==============================
Directions in Science
*TheScientificWorldJOURNAL*, (2007) **7**, 1193–1197
ISSN 1537-744X; DOI 10.1100/tsw.2007.212

TheScientificWorldJOURNAL
www.thescientificworld.com

### Syncytin and Cancer Cell Fusions

Lars-Inge Larsson*, Bolette Bjerregaard, Linda Wulf-Andersen and Jan Fredrik Talts

*Anatomy and Cell Biology, IBHV, Faculty of Life Sciences, University of Copenhagen, Gronnegaardsvej 7, DK-1809 Frederiksberg C, Denmark*

**E-mail:** lail@life.ku.dk

*Received July 13, 2007; Accepted July 19, 2007; Published August 17, 2007*

---

Cell fusions are important for embryonic and fetal development and for cell differentiation. Starting with the fusion between the egg and the sperm a number of subsequent fusions are important for the formation of a functional placenta as well as for muscle and bone development [1]. Moreover, many viruses use fusogenic envelope proteins to enter cells and expression of such proteins by the infected cells may lead to cell-cell fusions. Finally, while combating certain infections, macrophages may fuse to form giant cells[1].

Also malignant cells may fuse with each other as well as with normal host cells[2,3,4,5 and references therein] but the underlying mechanisms as well as the biological implications of such fusions are just being unravelled. In classical experiments, which became seminal to the discovery of tumor suppressor genes, it was observed that fusions induced to occur between cancer cells and normal cells led to suppression of tumorigenicity[6,7,8]. In contrast, other fusions, such as the ones between spleen cells and myeloma cells, did not lead to cell cycle arrest but resulted in monoclonal antibody-producing hybridomas with unlimited proliferative ability[9]. In yet other studies, fusions between e.g. melanoma cells and macrophages aggravated the malignant phenotype, underlining that the genetic make-up of the fusion partners dictated the outcome of the fusions[4,10,11]. This was recently further emphasized in studies by Duelli et al.[12] using cells with mutations in an oncogene or in p53. Additionally, it has been considered that cell fusions, which result in mononuclear hybrid cells (synkaryons) may result in increased genetic instability and, possibly, in malignant transformation[1].

During the course of studies of interactions between breast cancer cells and endothelial cells our group observed that such cells fused spontaneously to produce hybrids expressing chromosomal and protein markers characteristic of both parental lineages both *in vitro* and *in vivo*[3]. Initially, the hybrid cells were bi- or multinuclear with the parental genomes segregated in individual nuclei. However, they were capable of cell divisions and time course studies suggested that such divisions resulted in daughter cells expressing both parental sets of chromosomes in a single nucleus[3] (Fig. 1A). Studies of possible mechanisms underlying the fusions showed that the breast cancer cells expressed an endogenous retroviral envelope protein, syncytin-1, and that the endothelial cells expressed the corresponding D-type retroviral receptor (ASCT2)[13] (Fig. 1B, C).

A significant proportion of the human genome consists of retroviral sequences, which represent remnants of infections that occurred millions of years ago. Most of these sequences do not code for functional proteins due to mutations, insertions and deletions. However, syncytin-1 has been preserved during evolution, perchance reflecting its role in facilitating cell fusions creating chorionic gonadotrophin-producing syncytiotrophoblasts[14,15,16]. Syncytin-1 expression is highest in the placenta

*Corresponding author.
©2007 with author.
Published by TheSciemtificWorld: www.thescientificworld.com

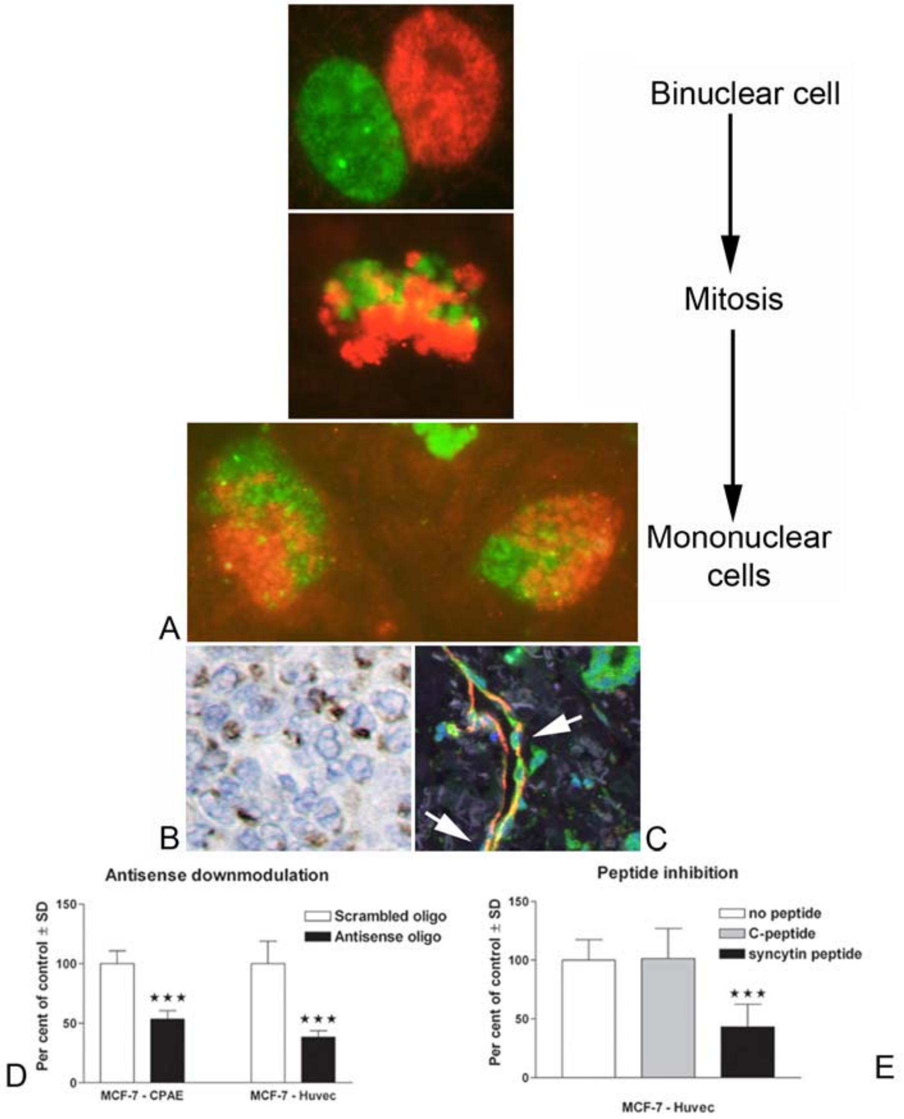

**FIGURE 1A**. Hybrids formed from fusions between human breast cancer cells and bovine endothelial cells reacted with fluorescent in situ hybridization (FISH) to reveal human chromosomes in red and bovine chromosomes in green. Initially, bi- (or multi-) nuclear cells form in which the parental genomes are segregated in different nuclei. Subsequently, mitoses, containing an admixture of human and bovine chromosomes occur and mononuclear daughter cells form. Interestingly, human and bovine chromosomes occupy different domains within these nuclei. **B.** Immunoperoxidase staining for syncytin-1 is present in numerous cancer cells in a ductal breast cancer. **C**. Double-staining for ASCT-2 (green immunofluorescence) and the endothelial cell marker CD31 (red immunofluorescence) in a breast cancer specimen shows colocalization in endothelial cells (arrows). In addition, ASCT2 expression is seen in adjacent tumor cells (green). **D.** Downmodulation of syncytin expression significantly decreases fusions between breast cancer (MCF-7) and bovine (CPAE) or human (HUVEC) endothelial cells. **E.** A syncytin inhibitory peptide significantly decreases fusions between human breast cancer cells and endothelial cells, whilst a control peptide has no effect.

and the protein uses ASCT2 and another amino acid transporter (ASCT1) as receptors[15,17]. The syncytin-1 gene is also expressed in testis, brain, breast and endometrial carcinomas[13,15,16,18,19]. A second related gene, syncytin-2 was recently also found to be fusogenic[20]. While the syncytin-1 gene is derived from the envelope-encoding gene of HERV-W, the syncytin-2 gene represents the envelope-encoding gene of another endogenous retrovirus (HERV-FRD)[20]. Syncytin-2 is expressed in the placenta[20] and may contribute to trophoblast cell fusions although solid evidence for such effects presently is limited to syncytin-1.

Syncytin-1 is expressed in man and in old world primates and is calculated to have entered the primate genome 25-40 million years ago[14]. However, in our very distant relatives, the rodents, trophoblasts also fuse to form syncytiotrophoblasts. Interestingly, two different retroviral envelope genes, syncytin-A and -B, are expressed in the placenta of all classes of muridae and may function in facilitating placental cell fusions in rodents[21], suggesting an astonishing example of parallel evolution due to accidental infections with unrelated retroviruses in primates and rodents.

Two lines of evidence suggest that syncytin-1 is also involved in breast cancer-endothelial cell fusions[13]. Firstly, antisense-mediated knockdown of syncytin-1 expression resulted in a highly significant inhibition of such cell fusions (Fig. 1D). Secondly, a syncytin-1 inhibitory peptide also strongly inhibited cell fusions (Fig. 1E). Interestingly, both approaches inhibited fusion efficiency by 40-50%. This may reflect that additional factors are involved. Interestingly, recent unpublished data from our laboratory show that also syncytin-2 is expressed by breast cancer cell lines. Additionally, recent studies show that cancer cells express syncytin-1 in 63 out of 165 (38%) of clinical breast cancer specimens and that both endothelial cells and tumor cells express ASCT2 [22]. This suggests that at least some of the molecular requirements for cancer-cancer and cancer-host cell fusions are present in clinical breast cancer. Syncytin-1 is also expressed by endometrial carcinomas and downmodulation of syncytin-1 expression almost totally inhibited endometrial carcinoma cell fusions[19]. Importantly, the latter study revealed that both estrogens and agents, which increases cAMP, upregulated expression of syncytin-1[19]. However, while cAMP-elevating treatments increased the frequency of cell fusions, estrogens did not. A possible explanation to this is that estrogens, but not cAMP-elevating agents, upregulated TGFbeta1. TGFbeta isoforms may be important negative regulators of cell fusion events. Evidence for this was that addition of TGFbeta 1 or 3 to cells treated with cAMP-elevating agents decreased cell fusions in both endometrial carcinoma cells and in trophoblast tumor (choriocarcinoma) cells and that immunoneutralization of TGFbeta increased the frequency of cell fusions in estrogen-treated cells[19]. These and additional data suggest TGFbeta isoforms to be important negative regulators of cell fusions and that they antagonize the effects of syncytin-1.

What is the role of syncytins in pathology? Expression of the gene has been implicated in multiple sclerosis and inefficient syncytin-1 expression may relate to placental malfunctions such as preeclampsia[18,23]. The data by others and us implicate syncytin-1 expression in tumor pathology[13,19,22]. Results by Strick et al.[19] showed that, in a small series of endometrial polyps and malignancies, the level of syncytin-1 expression correlated positively with malignancy. In contrast, our own studies of 165 human breast cancers showed that the level of syncytin expression correlated positively to improved disease-free survival of the patients [22]. It is yet too early to speculate on the mechanism behind this. Thus, although cancer-cancer and cancer-host cell fusions have been connected with syncytin-1 expression, this molecule possesses additional important roles in cell proliferation and immune modulation. Moreover, at least in glial cells, syncytin-1 upregulates expression of nitric oxide synthase (NOS) [18]. Importantly, NO production in tumor vessels has been linked to improved disease-free and over-all survival as well as to decreased metastatic potency in experimental systems[24,25,26]. Moreover, the effects of syncytin-1 on cell fusions is likely to be modulated by additional factors, one of which may be TGF-beta isoforms[19], and cell fusions may, as referred to above, produce cells of both increased and decreased malignancy. Finally, the specificities of existing syncytin antibodies tend to vary. It is probable that many antibodies also detect the closely related syncytin-2 protein and immunolocalizations in the placenta with different antibodies have produced contradictory results.

At the bottom line, it can be stated that syncytin-1 not only mediates trophoblast cell fusions but may also mediate cancer-cancer and cancer-host cell fusions. It may therefore be that syncytin-1 (and, perhaps, syncytin-2) is also involved in other cell fusions operating in a tightly controlled multiprotein network of facilitatory and inhibitory factors, which ensure that inapropriate cell fusions never occur in the healthy individual. The role of syncytins in tumor pathology is still uncertain – part of the effects may relate to cell fusion mechanisms while part may reflect effects of syncytins on immunomodulation, proliferation and expression of mediators like nitric oxide synthases and interleukin-beta.

### REFERENCES

1. Ogle, B.M., Cascalho, M., and Platt, J.F. (2005) Biological implications of cell fusion. *Nature Reviews Mol. Cell Biol.* **6,** 567-575.
2. Duelli, D., and Lazebnik, Y. (2003) Cell fusion: a hidden enemy? *Cancer Cell* **3**, 445-448.
3. Mortensen, K., Lichtenberg, J., Thomsen, P.D., and Larsson, L.-I. (2004) Spontaneous fusion between cancer cells and endothelial cells. *Cell. Mol. Life Sci.* **61**, 2125-2131.
4. Pawelek, J.M. (2005) Tumour-cell fusion as a source of myeloid traits in cancer. *Lancet Oncology* **6**, 988-993.
5. Goldenberg, D.M., Pavia, R.A., and Tsao, M.C. (1974) In vivo hybridisation of human tumor and normal hamster cells. *Nature* **250**, 649-651.
6. Harris, H., Miller, O.J., Klein, G., Worst, P., and Tachibana, T. (1969) Suppression of malignancy by cell fusion. *Nature* **223,** 363-368.
7. Wiener, F., Klein, G., and Harris, H. (1974) The analysis of malignancy by cell fusion. V. Further evidence of the ability of normal diploid cells to suppress malignancy. *J. Cell Sci.* **15,** 177-183.
8. Stanbridge, E.J. (1976) Suppression of malignancy in human cells. *Nature* **260,** 17-20.
9. Kohler, G. and Milstein, C. (1975) Continuous cultures of fused cells secreting antibody of predefined specificity. *Nature* **256,** 495-497.
10. Larizza, L., Schirrmacher, V., Graf, L., Pfluger, E., Peres-Martinez, M., and Stohr, M. (1984) Suggestive evidence that the highly metastatic variant ESb of the T-cell lymphoma Eb is derived from spontaneous fusion with a host macrophage. *Int. J. Cancer* **34**, 699-702.
11. Munzarova, M., Lauerova, L., and Capkova, J. (1992) Are advanced malignant melanoma cells hybrids between melanocytes and macrophages? *Melanoma Res.* **2**, 127-129.
12. Duelli, D.M., Hearn, S., Myers, M.P., and Lazebnik, Y. (2005) A primate virus generates transformed human cells by fusion. *J. Cell Biol.* **171**, 493-503.
13. Bjerregaard, B., Holck, S., Christensen, I. J., and Larsson, L.-I. (2006) Syncytin is involved in breast cancer-endothelial cell fusions. *Cell. Mol. Life Sci.* **63**, 1906-1911.
14. Mallet, F., Bouton, O., Prudhomme, S., Cheynet, V., Oriol, G., Bonnaud, B., Lucotte, G., Duret, L., Mandrand, B. (2004) The endogenous retroviral locus ERVWE1 is a bona fide gene involved in hominoid placental physiology. *Proc. Natl. Acad. USA* **101**, 1731-1736.
15. Blond J. L., Lavillette D., Cheynet V., Bouton O., Oriol G., Chapel-Fernandes, S. Mandrand, B., Mallet, F., and Cosset, F.L. (2000) An envelope glycoprotein of the human endogenous retrovirus HERV-W is expressed in the human placenta and fuses cells expressing the type D mammalian retrovirus receptor. *J. Virol.* **74**, 3321-3329.
16. Mi, S., Lee, X., Li, X., Veldman, G.M., Finnerty, H., Racie, L., LaVallie, E., Tang, X.Y., Edouard, P., Howes, S., Keith Jr,, J.C., and McCoy, J.M.(2000) Syncytin is a captive retroviral envelope protein involved in human placental morphogenesis. *Nature* **403**, 785-789.
17. Lavillette, D., Marin, M., Ruggieri, A., Mallet, F., Cosset, F.L., and Kabat, D. (2000) The envelope glycoprotein of human endogenous retrovirus type W uses a divergent family of amino acid transporters/cell surface receptors. *J. Virol.* **76**, 6442-6452.
18. Antony, J.M., van Marle, G., Opii, W., Butterfield, D.A., Mallet, F., Yong, V.W. Wallace, J.L., Deacon, R.M., Warren, K., and Power, C. (2004) Human endogenous retrovirus glycoprotein-mediated induction of redox reactants causes oligodendrocyte death and demyelination. *Nature Neurosci.* **7**, 1088-1095.
19. Strick, R., Ackermann, S., Langbein, M., Swiatek, J., Schubert, S.W., Hashemolhosseini, S., Koscheck, T., Fasching, P.A., Schild, R.L., Beckmann, M.W., and Strissel, P.L. (2007) Proliferation and cell-cell fusion of endometrial carcinoma are induced by the human endogenous retroviral syncytin-1 and regulated by TGF-beta. *J. Mol. Med.* **85**, 23-38.
20. Blaise, S., de Parseval, N., Benit, L., and Heidmann, T. (2003) Genomewide screening for fusogenic human endogenous retrovirus envelopes identifies syncytin 2, a gene conserved on primate evolution. *Proc. Natl. Acad. Sci. USA* 100, 13013-13018.
21. Dupressoir, A., Marceau, G., Vermochet, C., Benit, L., Kanellopoulos, C., Sapin, V., and Heidmann, T. (2005) Syncytin- A and syncytin-B, two fusogenic placenta-specific murine envelope gtenes of retroviral origin conserved in Muridae. *Proc. Natl. Acad. Sci. USA* 102: 725-730.
22. Larsson, L.-I., Holck, S., and Christensen, I.J. (2007) Prognostic role of syncytin expression in breast cancer. *Human Pathol.* **38**, 726-731.
23. Chen, C.P., Wang, K.G., Chen, C.Y., Yu, C., Chuang, H.C., and Chen, H. (2006) Altered placental syncytin and its receptor ASCT2 expression in placental development and pre-eclampsia. *BJOG* 113: 152-158.
24. Mortensen, K., Holck, S., Christensen, I.J., Skouv, J., Hougaard, D.M., Blom, J., and Larsson, L.I. (1999) Endothelial cell nitric oxide synthase expression in peritumoral microvessels is a favourable prognostic indicator in breast cancer *Clin. Cancer Res.* **5**, 1093-1097.
25. Mortensen, K., Christensen, I.B., Nielsen, H.J., Hansen, U., and Larsson, L.-I. (2004) A high frequency of peritumoral microvessels expressing endothelial cell nitric oxide synthase is a favorable prognostic factor in cancer of the colon. *Cancer Lett.* **216**, 109-114.
26. Qiu, H., Orr, F.W., Jensen, D., Wang, H.H., McIntosh, A.R., Hasinoff, B.B., Nance, D.M., Polypas, S., Qi, K., Song, C., Muschel, R.J., and Al-Mehdi, A.B. (2003) Arrest of B16 melanoma cells in the mouse pulmonary microcirculation induces endothelial nitric oxide synthase-dependent nitric oxide release that is cytotoxic to the tumor cells. *Am. J. Pathol.* **162**, 403-412.

**This article should be cited as follows:**

Larsson, L.-I., Bjerregaard, B., Wulf-Andersen, L. and Talts, J.F. (2007) Syncytin and cancer cell fusions. *TheScientificWorldJOURNAL* **7**, 1193–1197. DOI 10.1100/tsw.2007.212.
